# Epidemiology of *Mycobacterium bovis* Disease in Humans, the Netherlands, 1993–2007

**DOI:** 10.3201/eid1703.101111

**Published:** 2011-03

**Authors:** Christof J. Majoor, Cecile Magis-Escurra, Jakko van Ingen, Martin J. Boeree, Dick van Soolingen

**Affiliations:** Author affiliations: Academic Medical Center, Amsterdam, the Netherlands (C.J. Majoor);; Radboud University Nijmegen Medical Centre, Nijmegen, the Netherlands (C. Magis-Escurra, J. van Ingen, M.J. Boeree, D. van Soolingen);; National Institute for Public Health and the Environment (RIVM), Bilthoven, the Netherlands (J. van Ingen, D. van Soolingen)

**Keywords:** Mycobacterium bovis, M. tuberculosis disease, tuberculosis and other mycobacteria, epidemiology, Mycobacterium bovis, M. bovis, anti-TNF-α, extrapulmonary disease, gender differences, research

## Abstract

In the Netherlands, 1.4% of tuberculosis (TB) cases are caused by *Mycobacterium bovis*. After we admitted 3 patients with *M. bovis* infections to our reference hospital, we conducted a retrospective analysis of all *M. bovis* disease in the Netherlands during 1993–2007. We analyzed data from 231 patients for clinical, demographic, treatment, and outcome characteristics and for risk factors. Most patients were native Dutch (n = 138; 59.7%) or Moroccan (n = 54; 23.4%). Disease was mainly extrapulmonary (n = 136; 58.9%). Although 95 patients had pulmonary disease, person-to-person transmission did not occur, as shown by structural DNA fingerprinting analysis. Lymph node TB was more likely to develop in women (p<0.0001), whereas pulmonary *M. bovis* disease developed more frequently in men (p<0.0001). Diagnosis was accurate but delayed and led to inadequate treatment in 26% of the cases. Proportion of deaths from *M. bovis* disease was higher than that for *M. tuberculosis* disease.

*Mycobacterium bovis* disease was common in the Western world in the era before pasteurization of milk products. In 1938, the percentage of *M. bovis* disease among all patients with tuberculosis (TB) was 9% in Amsterdam and 11% in the rest of the Netherlands. In 1940, pasteurization became obligatory, and in 1952, the percentage of *M. bovis* disease had dropped to 1.5%–2.0% in Amsterdam (*1**,**2*). From 1945 until the mid-1960s, *M. bovis* infection was gradually eradicated in livestock in the Netherlands. Nevertheless, during 1973–1975, ≈2.5% of the human TB cases in the Netherlands were still caused by *M. bovis* (*3*). Since 1993, only 10 cases of *M. bovis* infection have occurred in livestock in the Netherlands. All were caused by infected livestock that had been imported (D. Bakker, pers. comm.). Because most *M. bovis* infections are contracted through the oral route, extrapulmonary manifestations were primarily observed.

Diagnosing extrapulmonary TB is generally difficult, and as the prevalence of TB has declined, the experience of physicians in diagnosing this specific infectious disease has also decreased, and therefore the time to diagnosis has increased (*4**,**5*). The difficulties in the diagnosis and treatment of *M. bovis* infections in 3 female patients at the University Centre for Chronic Diseases Dekkerswald prompted the present study. To investigate the magnitude of *M. bovis* infection in persons in the Netherlands, we conducted a retrospective study of patients with *M. bovis* infection and describe the epidemiologic, clinical, and bacteriologic findings.

## Materials and Methods

We retrieved data from 2 databases: one at the National Institute for Public Health and the Environment (RIVM), Bilthoven, the Netherlands, which contains the bacteriologic information of all *M. tuberculosis* complex isolates in the Netherlands. The other is the Netherlands Tuberculosis Register (NTR), an anonymous case register held by **KNCV** Tuberculosis Foundation in The Hague, the Netherlands. This database is based on voluntary registration but contains data from virtually all TB patients in the Netherlands.

The patients in the NTR database have been registered by their physicians. This register holds basic demographic, clinical, and some bacteriologic data. Death is registered as TB related or not TB related without further explanation. Treatment and treatment outcome are registered without further details. Exact treatment length could be determined because length was registered in 2 different categories. Therefore, we could categorize treatment length in 6 groups, namely <3 months, 4–5 months, 6 months, 7–9 months, 10–12 months, and >12 months.

We selected all *M. bovis* cases that occurred during 1993–2007. By using these 2 databases we had information on bacteriologic and clinical factors, demographic data, and risk factors, as well as treatment and outcome. We used the RIVM database for the bacteriologic data and localization of the infections and the NTR database for the demographic and clinical data, including treatment outcome. To distinguish *M. bovis caprae* and *M. bovis bovis* infections, we reviewed available data on IS*6110* restriction fragment length polymorphism, spoligotyping, and pyrazinamide susceptibility for all *M. bovis* isolates analyzed in this study (*6**,**7*). Because IS*6110* typing of all *M. tuberculosis* complex isolates is routinely performed in the Netherlands, we also analyzed information on possible interhuman transmission of *M. bovis*.

Associations between localization of infection in the patient, geographic distribution, ethnicity, etc. were evaluated on basis of information from both databases by using the χ^2^ test. Mortality rates were evaluated by using the NTR database and were correlated with the demographic and clinical data.

## Case Reports

### Patient 1

A 73-year-old woman sought treatment for nonproductive cough and dyspnea. Medical history included severe rheumatoid arthritis, for which she had received treatment with anti–tumor necrosis factor-α (TNF-α) for 1 year. High-resolution computed tomography (CT) imaging of the thorax showed micronodular lesions in both lungs. CT imaging of the brain indicated several cerebral nodular lesions, suspected to be tuberculomas. Culture of bronchoalveolar lavage fluid yielded *M. bovis*. The patient was treated with isoniazid, rifampin, and ethambutol. Moxifloxacin and steroids were added, because of the high penetration level through the blood–brain barrier of moxifloxacin and to compensate for the absence of pyrazinamide in the treatment regimen.

### Patient 2

**Figure 1:**
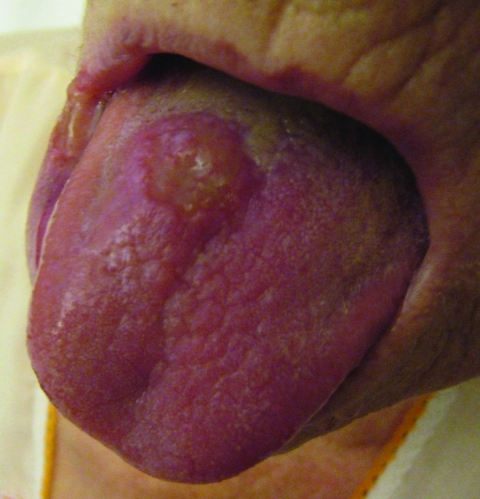
*Mycobacterium bovis* lesion on the tongue of patient 2, the Netherlands.

An 84-year-old woman with a medical history of myelodysplastic syndrome and diabetes mellitus type 2 sought treatment for a traumatic leg wound that did not heal, and her condition gradually progressed to generalized skin lesions. She was referred to the dermatologist in our hospital. Culture of a skin biopsy specimen grew *M. bovis.* The patient had lesions on her tongue, typical of TB (Figure 1), and on her the larynx and in her lungs. The patient’s condition resolved after 9 months of treatment with isoniazid, rifampin, and ethambutol. Contact tracing showed no human transmission.

### Patient 3

An 87-year-old woman with severe rheumatoid arthritis sought treatment for weight loss, cough, night sweats, and dyspnea 1 year after starting anti–TNF-α treatment. Radiologic imaging showed a single nodular lesion of the lung parenchyma and pleural fluid. Culture of a bronchoalveolar lavage specimen yielded *M. bovis*. The patient received isoniazid, ethambutol, and moxifloxacin for 18 months because she had an intolerance for rifampin. No contact tracing was performed**.**

## Results

**Figure 2:**
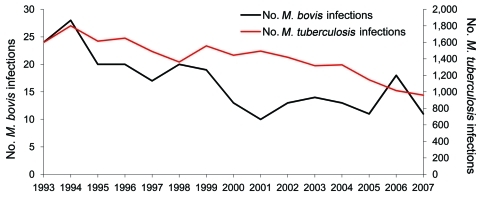
*Mycobacterium tuberculosis* and *M. bovis* infections, the Netherlands, 1993–2007. Data derived from the National Institute for Public Health and the Environment (RIVM) database.

**Figure 3:**
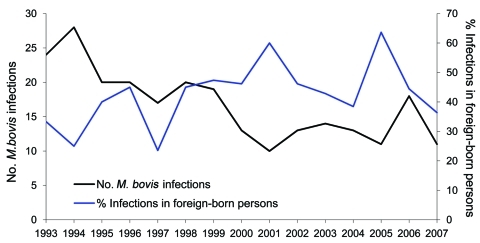
Percentage of *Mycobacterium bovis* infections in foreign-born persons and total number of *M. bovis* infections, the Netherlands, 1993–2007. Data derived from the National Institute for Public Health and the Environment (RIVM) database.

During 1993–2007, a total of 16,059 patients were registered in the NTR with culture-proven TB; 231 (1.4%) patients were registered with *M. bovis* infection (*8*) (Figure 2). The number of patients with *M. bovis* infection born outside the Netherlands (foreign-born) showed an increase in the proportion from 35.1% to 46.3% in the 1ast 5 years of the study period (Figure 3). Over time, the percentage of patients with *M. bovis* TB who live in major cities increased from 33.3% to 41.8%, with an accompanying decline elsewhere in the Netherlands. No relation was found between outbreaks in livestock and human disease.

**Figure 4:**
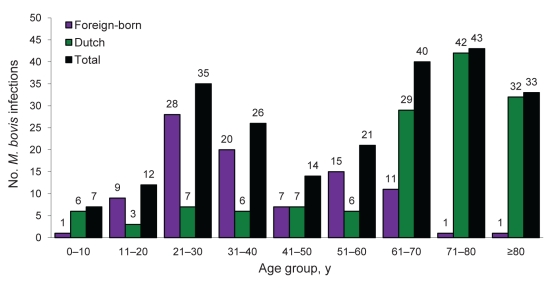
Number of *Mycobacterium bovis* infections, according to patient age and origin, the Netherlands, 1993–2007. Data derived from the National Tuberculosis Registry database.

Among patients with *M. bovis* disease, a significant difference in mean age was noted among those born in the Netherlands and immigrants. In native Dutch persons with *M. bovis* disease, the median age was 64.8 years, whereas among immigrants, the median age was 38.6 years (Figure 4). We observed 58 cases in young foreign-born patients and 16 cases in young Dutch patients (<40 years). Five Dutch patients had a general risk factor for contracting *M. tuberculosis* complex disease (1 with drug addiction, 1 with alcohol addiction, and 3 with risk factors not specified) and 44 foreign-born had 1 or 2 general risk factors (32 immigrants, 8 with fugitive status, 3 illegal immigrants, and 1 TB contact, 1 in criminal detention, 1 with risk factor not specified).

Most of the patients were native Dutch (138 [59.7%] of 231). Most foreign-born patients came from Morocco (54 [23.4%] of 231). The other immigrants had other places of origin: Europe (n = 6; 2.6%), Africa (n = 15; 6.5%), Asia (n = 9; 3.9%), and South America (n = 3; 1.3%) (Table 1). A distinction between native Dutch and second-generation Dutch immigrants could not be made.

**Table 1:** Clinical and demographic data for *Mycobacterium bovis* TB case-patients, by sex, as recorded in the Netherlands Tuberculosis Registry, the Netherlands, 1993–2007*

Characteristic		Dutch, no. (%), n = 138	Foreign-born, no. (%),† n = 93	Total, no. (%), N = 231
All case-patients				
M		56 (40.6)	49 (52.7)	105 (45.5)
F		82 (59.4)	44 (47.3)	126 (54.5)
Age, y				
0–60				115 (49.7)
M		13	45	
F		22	35	
>60				116 (50.3)
M		43	4	
F		60	9	
Localization				
Pulmonary				80 (34.6)
M		26	25	
F		22‡	7§	
Respiratory tract				19 (8.2)
M		5	5	
F		8	1	
Meningeal and CNS				6 (2.5)
M		2	0	
F		F	0	
Intestinal tract				13 (5.6)
M		0	3	
F		5	5	
Bone and joint				12 (5.2)
M		4	2	
F		5	1	
Urogenital tract				13 (5.6)
M		4	3	
F		4	2	
Other organs (e.g., lymph nodes)				59 (25.5)
M	5		9	
F	24¶		21#	
Miliary TB				11 (4.8)
M	6		0	
F	3		2	
Unknown				18 (7.8)
M	4		2	
F	7		5	

*TB, tuberculosis; CNS, central nervous system. †Foreign-born patients originated from other European countries (n = 6), Africa (n = 69), Asia (n = 9), South America (n = 3), and unknown areas (n = 6). ‡p = 0.018. §p<0.001. ¶p = 0.004. #p = 0.002.

*M. bovis* TB mainly had appeared as extrapulmonary disease; 136 (58.9%) of 231 patients had extrapulmonary disease alone, 68 (29.4%) of 231 had pulmonary *M. bovis* disease, and 27 (11.7%) of 231 had both pulmonary and extrapulmonary disease. A significant difference between men and women was found in terms of localization of disease. In women, lymph node TB was more likely to develop, mainly in the cervical region (p<0.0001); men more often had pulmonary *M. bovis* disease (p<0.0001). This finding was seen in all age groups in both Dutch and foreign-born patients. (Table 1)

Several treatment schedules were used for our study population; 157 of 231 patients received a combination of isoniazid, rifampin, ethambutol, and pyrazinamide, or another pyrazinamide-containing antimicrobial drug combination. Treatment with pyrazinamide-containing regimens had no negative effect on treatment outcome.

According to World Health Organization (WHO) standards, a regimen of isoniazid and rifampin for 9 months and ethambutol for 2 months is indicated for treating *M. bovis* disease. In our study population, we noted that 113 (52%) of 217 patients were cured and completed treatment according to the WHO standard. Thirty-seven (17%) of 217 patients were considered cured with an insufficient treatment schedule, duration, or both. For 14 patients, outcome data were not available. No significant associations were found between insufficient treatment and particular disease localizations or outcome.

An anonymous questionnaire distributed to the physicians who were caring for the patients at the time of disease (34 sent and 24 returned) showed that most of the physicians followed the Dutch TB guidelines, which are based on the WHO standard. A problem these colleagues faced was the time lag between sampling and the results of culture, identification, and drug susceptibility tests, which sometimes took as long as 4 months.

Treatment outcome comparisons for Dutch and foreign-born patients in the Netherlands are shown in Table 2. The overall proportion of deaths for the infection itself was 5.2% for *M. bovis* disease, and the proportion of deaths associated with non–TB-related causes (e.g., cardiac disease and hematologic malignancy) for patients with *M. bovis* disease was 14.7%.

**Table 2:** Treatment results according to age, sex, and localization of *Mycobacterium bovis* disease in Dutch and foreign-born case-patients, as recorded in the National Tuberculosis Registry, the Netherlands, 1993–2007*

Variable	No. (%) Dutch patients, n = 138					No. (%) foreign-born patients, n = 93			
	Treatment completed	Cause of death		Other†		Treatment completed	Cause of death		Other†
		TB-related	Non–TB-related				TB-related	Non–TB-related	
Total	85 (61.6)	10 (7.2)	28 (20.3)	15 (10.9)		66 (71.0)	2 (2.1)	6 (6.5)	19 (20.4)
Age, y									
0–60	25	1	3	6		59	2	3	16
>60	60	9	25	9		7	0	3	3
Sex									
M	33	3	18	2		35	0	3	11
F	52	7	10	13		31	2	3	8
Localization									
Pulmonary	26	2	9	3		21	0	2	5
Extrapulmonary	52	5	11	10		44	0	3	11
Pulmonary and extrapulmonary	7	3	8	2		1	2	1	3

*TB, tuberculosis. †Treatment not completed or treatment continued elsewhere.

An analysis of the overall deaths from bovine TB according to sex, age, ethnicity, and disease localization is shown in Table 3. A correlation was found between death and age >60 years, Dutch nationality, and miliary disease. When dividing deaths into the categories of TB related and not–TB related, only high age was statistically significant between the groups; all other variables were not significant (Table 4). Cause and rate of death differed between sexes: among women, death was more often related to *M. bovis* TB; in men, the overall mortality rate was higher, although these differences were not statistically significant.

**Table 3:** Correlation of overall deaths from *Mycobacterium bovis* disease with demographic variables, the Netherlands, 1993–2007*

Variable	p value
Sex	0.31
Age >60 y	<0.0001
Dutch nationality	0.001
Disease localization (miliary TB)	<0.0001

*χ^2^ test. TB, tuberculosis.

**Table 4:** Correlation of TB-related and non–TB-related deaths according to demographic variables, the Netherlands, 1993–2007*

Variable	p value
Sex	0.58
Age >60 y	0.03
Dutch nationality	0.91
Disease localization (miliary TB)	0.49

*χ^2^ test. TB, tuberculosis.

## Discussion

*M. bovis* TB comprised 1.4% of all TB cases in the Netherlands during 1993–2007. This finding is comparable to those of studies in other countries where control and corresponding control efforts of *M. bovis* TB in livestock are present (*9**–**11*). During 1993–1997, mainly elderly Dutch persons were found to have bovine TB; later (1998–2007), the infections were divided more equally between the native Dutch and immigrants (Figure 3).

*M. bovis* TB in the population of the Netherlands shows an age curve with double peaks. The younger patients were mostly foreign-born or first- and second-generation immigrants, who may have (frequently) traveled back to their country of origin or contracted an *M. bovis* infection by the oral route before coming to the Netherlands. These patients more often had extrapulmonary *M. bovis* disease, which mainly affected the cervical or abdominal lymph nodes. Ingestion of unpasteurized dairy products is the most likely route of infection (*12**,**13*). The 12 Dutch patients, ages 20–40 years, may have been in contact with nonpasteurized food while traveling to developing countries because the chance of contracting a *M. bovis* infection in the Netherlands is considered low.

The second age peak contains elderly native-born Dutch persons. This result is most likely a birth-cohort effect because these persons most probably had a late endogenous reactivation of latent *M. bovis* infection contracted in the era before pasteurization of milk was introduced and while *M. bovis* infection in livestock was still highly prevalent in the Netherlands. Spoligotyping of their isolates showed a single typical pattern, which is considered the old predominant cattle-endemic strain of the Netherlands (*14*). This epidemiologic trend has also been seen in other European countries, including Norway, Sweden, and Belgium (*9**,**10*).

Endogenous reactivation of *M. bovis* infections in elderly patients follows impairment of immunity from hematologic causes, immune modulation by medication (including anti–TNF-α treatment), other coexisting conditions, or immunosenescence. As the use of anti–TNF-α treatment rises, due to increasing indications of rheumatologic and gastrointestinal diseases, mycobacterial disease will likely become more common (*15*). Two of the patients we described had negative tuberculin skin test (for *M. tuberculosis* complex) and chest radiograph results and, therefore did not receive isoniazid prophylaxis before they received anti–TNF-α treatment. This result calls into question the efficacy of the screening protocol used in the Netherlands to evaluate patients before they receive anti–TNF-α treatment. Of note, reactivations of latent TB usually occur in the first 3–4 months of anti–TNF-α therapy (*15*), although in our 2 *M. bovis* patients, reactivation occurred after 1 year. This long-term asymptomatic reactivation of disease has been previously observed (*16**,**17*) and raises questions involving the role of the bacteriologic virulence factor in the pace of the reactivations.

In the Netherlands, the 2 largest immigrant populations are from Turkey and Morocco (378,330 and 341,528 persons, respectively) (*18*). A high occurrence of *M. bovis* disease was observed in Moroccan patients, whereas, to our surprise, *M. bovis* disease was rare in Turkish patients. Both populations come from agricultural areas where pasteurization of dairy products is not common. Popular raw milk cheeses in Morocco (*jben*) and Turkey (*kasar, tulum*) have been found to contain *Listeria* and *Brucella* species in as many as 8.2% of the samples tested (*19**,**20*). In both studies, cheeses were not tested for *M. bovis*. However, *M. bovis* can survive in raw milk cheese and cause an infection after it is eaten, as was described recently for a cluster of infections resulting from consumption of fresh Mexican cheese in New York and San Diego (*12**,**13**,**21*). Besides consumption of fresh cheese and unpasteurized milk, consumption of raw or undercooked meat is also a possible route of oral transmission (*10**,**22**,**23*).

More women than men were infected by *M. bovis*, contrary to the epidemiology of *M. tuberculosis*. Other studies about *M. bovis* do not show this result (*9**,**11*). This difference could be an age-related effect for Dutch patients; however, one can also see this difference in the Moroccan patient population (data not shown).

Notably, we also found a difference in disease localization between men and women. More cervical lymph node TB, which was not correlated with age, was diagnosed for female patients. Female sex has in general been considered a risk factor for *M. tuberculosis* extrapulmonary TB (*24**–**26*). However, this finding has not been described for *M. bovis* TB. Various explanations have been given for this gender difference in TB. One possible explanation could be a difference in the immunity to *M. tuberculosis* complex infection. Several studies have been conducted in humans to compare the immune response in both sexes. Differences in reaction among others in TNF and interleukin-10 production have been found (*27**,**28*). Another explanation for this finding may be related to the route of infection. Men could be more likely to become infected through the tracheal route by aerosols from diseased cattle, whereas women become infected by ingesting *M. bovis* while preparing or consuming contaminated food. Lastly, smoking has also been identified as a risk factor for pulmonary TB (*25*), which (assuming a higher percentage of men are smokers) could explain the difference we found in our study that men have more pulmonary *M. bovis* disease than women. Unfortunately, we could not retrieve smoking status from the databases.

The geographic distribution of *M. bovis* TB in the Netherlands showed a proportional increase in *M. bovis* infections in the major cities in the western part of the Netherlands during 1993–2007. This finding probably reflects the population demographics of the Netherlands, where most persons live in and between major cities, but also by the facts that immigrants mainly live in major cities and that the elderly persons who contracted *M. bovis* TB in the prepasteurization era are undergoing a natural decline in health.

We showed that, in 37 (17.1%) of the 217 patients, treatment of *M. bovis* disease cases in this study was not compliant with international guidelines. Although all *M. tuberculosis* complex isolates in the Netherlands undergo drug susceptibility testing, apparently the results of such testing do not reach clinicians. Recognition of *M. bovis* as the causative agent of TB does not in itself lead to the conclusion that adjustment of the therapy is required; many physicians only make adjustments after receiving the results of drug susceptibility testing, which usually arrive quite late because of the slow growth of *M. bovis*. Moreover, the quick molecular identification of cultured *M. bovis* still implies a culture delay of ≈4 weeks. Better education of physicians and increasing future application of direct molecular identification of *M. bovis* from clinical samples, i.e., sputum or lymph node aspirates**,** is therefore needed.

The overall proportion of deaths among patients with *M. bovis* disease was higher (19.9%) than that among patients with *M. tuberculosis* disease (4.4%) in the Netherlands. The mortality rates during 1993–2007 were 5.2% for *M. bovis* disease versus 1.9% for *M. tuberculosis* disease. This result is probably related to the higher prevalence of miliary and central nervous system localization of *M. bovis* disease. Death rates from other, non–TB-related, causes for patients with *M. bovis* infection were 14.7% compared with 2.5% for *M. tuberculosis* infection (*8*). Subgroup analysis of *M. bovis* showed that the proportion of deaths was higher among Dutch than among foreign-born patients, but this result most likely correlates with age and impairment of immunity (*10**,**11**,**29*).

We cannot explain the unexpected trend in lower survival of female patients after an episode of *M. bovis* disease. Previous studies have shown that male sex is a risk factor for unsuccessful treatment (*30**,**31*), but the numbers in our study were too small to draw any firm conclusions.

This study had limitations. First, the analysis of the results was hampered by the relatively low number of foreign-born patients (n = 93) and the heterogeneous nature of the study population. Therefore, only data concerning patients from Morocco (n = 54) could be analyzed separately, and differences with the Dutch case-patients were found in terms of age, mortality rate, and primary localization of disease.

Another limitation is that a distinction could not be made between native Dutch and second-generation immigrants born in the Netherlands. This lack of distinction implies that some patients have roots in a foreign country where the risk for *M. bovis* TB could be higher.

In conclusion, the incidence of *M. bovis* disease in the Netherlands is comparable to that in other countries in which control programs for *M. bovis* infection are enforced. Gender differences in clinical features and mortality rates were found in our cohort of patients. The disease now mainly infects immigrants from Morocco and elderly Dutch citizens. Anti-TNF-α treatment is an emerging cause of endogenous reactivation of *M. bovis* disease in elderly Dutch patients, as occurred in 2 of the recent bovine TB cases described in this article; reactivation may be slower than for *M. tuberculosis* infection.
